# Skin cancer awareness and sun protection advice for outdoor workers in Australia: Review of communication channels to optimise reach and relevance

**DOI:** 10.1177/20552076251404498

**Published:** 2026-01-19

**Authors:** Christina Verma, Jessica Lehane, Monika Janda

**Affiliations:** 1 Centre for Health Services Research, Faculty of Medicine, 1974 The University of Queensland, Queensland, Australia; 2 Institute of Health and Biomedical Research, School of Public Health and Social Work, Faculty of Health, Queensland University of Technology, Queensland, Australia

**Keywords:** Health communication, digital technology, skin cancer, public health, digital health intervention

## Abstract

**Aim:**

Skin cancer incidence in Australia is among the highest in the world, particularly for outdoor workers, and requires, clear and relevant health promotion resources. Government and non-government organisations across Australia play a key role in providing information and advice on how skin cancer awareness and sun protection advice should be promoted to at-risk population groups like outdoor workers. This study reviewed online resources developed by key organisations around recommended methods for communicating skin cancer awareness and sun protection messages to outdoor workers/outdoor workplaces.

**Methods:**

A rapid desktop review was undertaken in April 2021, focusing on publicly available online resources developed by federal government, state governments and cancer councils and available on websites in Australia. Resources included were selected if they addressed skin cancer, skin cancer prevention, sun safety or sun protection for outdoor workplaces and/or outdoor workers. Selected resources were re-reviewed in November 2024 to compare for changes.

**Results:**

Twenty-five resources were included in this review. Most (19/25) provided advice about how to communicate skin cancer awareness/sun protection messages to outdoor workers, while some did not (4/25). Use of digital technology to reach outdoor workers with sun-safe messages was mentioned in 12/25 resources. These resources mainly encouraged the use of the SunSmart Ultraviolet (UV) mobile application (8/25), specific sun-safety websites (3/25) and/or participation in an online course (2/25). Resources also mentioned non-digital ways of communicating information to outdoor workers.

**Conclusion:**

Digital technology is increasingly being used to communicate skin cancer prevention-related messages to outdoor workers, but non-digital ways are still more common. Increasing awareness about the effectiveness of digital health interventions in skin cancer prevention via online resources is essential for future-proofing sun-safety uptake in contemporary outdoor workplaces.

## Introduction

Two out of three Australians are expected to develop skin cancer by age 70.^1^ Ultraviolet (UV) radiation is the leading cause of skin cancer and increased exposure to UV radiation initiates and promotes tumours.^2^ In Australia, high-risk population groups like outdoor workers are exposed to high levels of UV for a significant portion of their day due to the nature of the work they undertake.^3–6^ A person can be considered an outdoor worker if they work under the sun for over 3 h a day in industries like construction, transport, building, utilities, agriculture and service.^7^ It has been estimated that approximately 37% of males and 8% of females are regularly exposed to UV radiation at work – this equates to up to 2 million workers in Australia.^8^ Furthermore, studies have shown that less than 9% of outdoor workers are fully protecting themselves from UV radiation while working.

The increasing burden of skin cancer remains a great challenge in the Australian workforce. Skin cancer-related illnesses, and related compensation claims made by outdoor workers have had major impact on the Australian economy.^9^ Between 2000 and 2012, there were 1970 workers who claimed compensation for sun-related injury/disease in Australia, at a total cost of $63m in compensation payments.^10^ To stem the rising incidence of skin cancer in outdoor workers, the Australian Government has enforced rules and regulations around employers’ responsibility towards decreasing the hazard of UV radiation along with the health ramifications for this population group. Particularly, employers in Australia are required to communicate and support health-promoting sun protective behaviours to outdoor workers to decrease their risk of UV-related skin damage and skin cancer incidence.^11^

Evidence suggests that educational and workplace policy interventions can be effective methods of changing sun protection behaviour and managing UV exposure risk during work.^7,12–14^ However, outdoor workers are a very mobile workforce that may not attend the headquarters frequently. Digital technologies such as mobile applications, text-messaging interventions, videos and other online resources are increasingly considered to be useful communication channels for conveying health promotion messages in such situations.^15,16^ Digital health interventions can be defined as those that deliver health promotion messages through electronic devices, such as mobile phones; use software, such as computer programs or apps or provide education or advice via websites.^17^ There is growing evidence around the use of digital health interventions to communicate best practice health advice.^18–20^ Utilising digital health interventions to promote sun-safety behaviour to outdoor workers may contribute to greater awareness, uptake of sun-safety behaviours and eventually lower rates of skin cancer.

Governments and peak non-government bodies across Australia have a critical role to play in alleviating the growing impact of skin cancer in communities by providing evidence based, clear recommendations to workplaces on how to promote effective skin cancer prevention interventions for the immediate and long-term safety and wellbeing of their outdoor workers. However, it is unknown if existing resources developed by government and non-government organisations to promote sun safety have been successfully translated into digital interventions to increase awareness and uptake of these interventions. Without increasing reach through digital communication, outdoor workplaces and outdoor workers are at risk of not receiving advice relating to awareness and prevention of skin cancer in a technology-driven world.

As a preliminary scoping activity to a larger body of research, this study aimed to review online resources developed by the federal government, state governments and cancer councils across Australia to identify their recommendations regarding the use of digital technology in communicating sun protection and skin cancer awareness to outdoor workers/outdoor workplaces. Through this rapid desktop review, readers will be able to gain valuable insights about where current gaps exist and where further research is required.

## Methods

A rapid desktop review was undertaken by two reviewers in April 2021 of publicly available sun exposure/sun protection educational resources. The resources were cross-checked by one reviewer in November 2024 to see whether any information was updated in the resources. The review was restricted to online resources developed by federal government, state governments and cancer councils in Australia, which were available on the internet. Analysis was restricted to these organisations due to their wide-reaching authoritative influence on preventive health decisions in Australia and likelihood to be referenced by workplaces when developing their own sun protection policies, procedures and messaging. For the purposes of this study, we defined a resource as an online policy, recommendation, guide, position statement, policy template, information webpage, online report or pamphlet.

For the purpose of this review, the term ‘communication channel’ was defined as the medium used to reach a particular population group.

## Review process

A rapid desktop review was undertaken by two independent reviewers in April 2021. A comparison review was undertaken in 2024 by one reviewer to identify whether any additional recommendations were provided against the topics of interest. Terms used for the Google search were ‘sun exposure’ AND/OR ‘sun protection’ AND/OR ‘sun’ paired with the term ‘policy’ AND/OR ‘resource’ AND ‘outdoor worker’ OR ‘outdoor workplace’.

Online resources included were selected based on their inclusion of general skin cancer information and/or; general skin cancer prevention and/or; sun safety or sun protection and; be specific to either outdoor workplaces and/or outdoor workers. This review followed a similar protocol to a recent review undertaken to understand the benefits and risks of UV radiation.^21^

The review was undertaken in two parts:

- The first part of the review identified whether online resources provided information about:
  - communicating skin cancer prevention/sun protection messages;
  - communicating such messages through digital technology;
  - the evidence of digital health interventions in skin cancer prevention;
  - the importance/role of outdoor workplaces in raising awareness of skin cancer/sun protection to their workers; and
  - the need for workplaces to adopt sun protection measures for the safety of outdoor workers.
- The second part of the review identified whether the online resources provided specific recommendations regarding the type/s of digital and non-digital communication channels that should be used to communicate skin cancer awareness and sun protection messages to outdoor workers.

Quality and consistency of the review was maintained by both reviewers using the same extraction template and ensuring that resources were readily accessible.

## Data analysis

The two independent reviewers read all of the online resources and undertook the review in the two parts outlined above. For the first part of the review, the reviewer identified whether online policies provided advice in relation to communicating skin cancer awareness to outdoor workers and compiled data into a table to identify the proportion of resources that provided these types of advice. This data was grouped by organisation type (federal government, state governments and cancer councils) and summarised in tabular form. Total number of organisations that provided the advice was calculated to identify trends. The main dataset established in 2021 remained unchanged.

## Results

The search initially identified over 29 weblinks to resources specific to skin cancer prevention/sun protection for outdoor workers/outdoor workplaces. However, four weblinks did not meet the inclusion criteria and therefore were excluded from the review.

A total of 25 resources^11,22–45^ remained and were eligible for review. Three resources were developed by federal government,^22–24^ nine resources by state governments^25–32^^,44^ and 13 by cancer councils across Australia.^11,33–43^^,45^ Resources included PDF guides, PDF brochures, factsheets and information web pages for outdoor workers/outdoor workplaces.

A comparison to the same resources in 2024 did not show any updates to the advice or recommendation provided.

### Key findings

All 25 resources provided factual information regarding skin cancer, for example, information on how UV overexposure may cause skin cancer and what skin cancer may look like, and skin cancer prevention and sun-safety information. None of the resources (0/25) provided the evidence around the benefits of using digital health interventions to promote and support skin cancer prevention. Most of the resources (19/25) provided general advice about communicating sun cancer awareness/sun protection messages and advice to outdoor workers through analogue channels such as posters, pamphlets and so on. Very few resources provided advice about communicating skin cancer awareness/sun protection messages through digital technology such as mobile apps or text messaging (9/25). Most resources commented on the need for workplaces to adopt sun protection measures for outdoor workers (23/25), and the important role of outdoor workplaces in raising skin cancer prevention awareness amongst their workers (21/25).

Of the 25 resources, 19 resources provided information about a communication channel, for example, seminars and posters and while six resources did not. A total of 12/25 resources identified/gave examples of digital communication channels that could assist outdoor workplaces/outdoor workers in understanding more about skin cancer and sun-safe activities. Of these 12 resources, 8/12 referred to the use of the cancer council's SunSmart UV app and its digital alert functions, which could be used to remind outdoor workers about the times sun protection is needed depending on their location. A few resources (3/12) mentioned UV index websites as a mechanism to communicate sun protection-related information, while some other resources (7/12) mentioned the use of mobile phones as a mechanism to accessing UV-related information including the use of mobile applications. One resource in particular noted that staff could receive sun-safety-related SMS messages from their employers in efforts to improve sun protection behaviour. Two resources also recommended the use of an online ‘UV and Heat Awareness’ training course for outdoor workers.

A total of 15/25 resources gave examples of non-digital communication channels that assist in raising skin cancer awareness and sun protection messages. These resources mainly identified training sessions (11/25). Two resources noted the use of posters, while four resources noted the implementation of sun exposure policy documents as a communication tool. Some resources (7/25) referenced both digital and non-digital communication channels. Table 1 provides an overview on the types of advice the 25 resources provide.

**Table 1.** Advice relating to communication of skin cancer awareness messages in outdoor worker sun protection/sun exposure resources developed by federal government, state governments and cancer council organisations.^a^

First part of review: Advice relating to communication of skin cancer awareness messages through outdoor worker sun protection/sun exposure resources	Federal government (*n* = 3)	State governments (*n* = 9)	Cancer councils (*n* = 13)	Total (*n* = 25)
Evidence of digital health interventions benefits in skin cancer prevention	0/3	0/9	0/13	0/25
Communicating skin cancer awareness/sun protection messages/advice	1/3	9/9	9/13	19/25
Communicating skin cancer awareness/sun protection messages/advice through digital technology	1/3	4/9	4/13	9/25
Importance/role of outdoor workplaces in raising awareness of skin cancer/sun protection to their workers	2/3	7/9	12/13	21/25
Need for workplaces to adopt sun protection measures for the safety of outdoor workers	3/3	8/9	12/13	23/25
Second part of review: Advice about the types of communication channels recommended/identified in resources regarding raising skin cancer awareness and sun protection messages to outdoor workers	Federal government (*n* = 3)^b^	State governments (*n* = 9)^b^	Cancer councils (*n* = 14)^c^	Total (*n* = 25)^d^
Digital communication channel	1/3	2/9	9/13	12/25
Non-digital communication channel	2/3	4/9	9/13	15/25

a Contact the corresponding author for detailed table on findings and references for resources. ^b^Note that one resource of the total 25 resources did not provide any information in relation to this. ^c^Note that two resources of the total 25 resources did not provide any information in relation to this. ^d^Note that four of the total 25 resources did not provide any information in relation to this.

## Discussion

As society moves to a more digitised world, understanding the role digital technology plays in disease prevention is critical. It is crucial that policy makers and policy influencers harness the potential of digital technology to improve skin cancer outcomes.^46^ Digital technology is a particularly effective tool in communicating information that provides trusted information, raises awareness of disease risk and supports protective behaviours and when necessary behavioural change. Behaviour change outcomes can include improved sun protection, for example use of sunscreen or protective clothing applications, and a culture of sun safety becoming a normative behaviour in a workplace.^46^ Evidence suggests that advocating for the use of digital resources for communicating sun protection messaging to outdoor workers assists with increasing the uptake of these behaviours.^47^

While this review demonstrates that there is some mention of using digital communication in existing resources, there is an opportunity for online resources developed by Australian government and non-government organisations to provide more information on how digital interventions can be utilised in outdoor workplaces to support staff sun safety. In addition to mentioning that sun protective messaging can be used, in the future, online resources on government, cancer councils and trusted other organisations should be updated to provide support for workplaces on how to implement such digital tools. This will enable outdoor workplaces to make an informed choice about which intervention to use to promote sun-safety behaviour in workers.

However, when providing information about best practice interventions, policy makers and subject matter experts should also provide references to evidence that note the success of the intervention. Of the nine resources that did recommend this use of digital communication methods, none of these resources provided evidence of the success of digital health interventions in increasing sun-safety knowledge, attitudes or behaviours or the even more important long-term goal of decreasing skin cancer rates in Australia. Inclusion of evidence endorsing the use of digital communication methods can have a direct impact on encouraging the uptake of digital communication modes.^48^ Differences in the number of recommendations provided by cancer councils and federal and state governments were also observed in resources, calling for greater consistency in how recommendations are reported.^21^

Although this review showed that the commonly recommended strategies of communicating sun protection messages in Australia were through the implementation of educational sessions and general sun protection policy in workplaces, these analogue strategies may have a smaller reach and lower compliance than digital communication strategies, as they are static, often once off and not adaptable to a specific work setting or worker. An opportunity exists for policy makers in government and non-government organisations across Australia to widely promote approaches that could future-proof strategies for digital delivery and make a substantial impact on skin cancer prevention.^49^

### Future research

This study provides insight into some key gaps that require further exploration. As digital communication capabilities continue to evolve and expand as a key global solution to disease prevention, there needs to be a greater focus of promoting its use and evidence particularly in addressing diseases like skin cancer in high-risk population groups in Australia. Policy makers and influencers from government and non-government organisations have a key role to play with this. Understanding the factors that can help increase uptake and use of digital health intervention in outdoor workplaces through a survey study within the Australian context can also assist policy makers and influencers to shape recommendations that encourage use of digital communication in delivering skin cancer awareness advice for this high-risk population group. An opportunity also exists for policy makers in government and non-government peak bodies to collaborate and develop a united position on reporting best practice recommendations for consumers^21^ particularly in relation to using digital technology as a tool to reach outdoor workers. Collaboration can also extend to designing and implementing approaches on how resources can be promoted by government and non-government organisations unitedly to improve reach to consumers.

## Limitations

This rapid review used publicly available websites and was undertaken following best practice guidelines with extraction by two independent researchers. A more comprehensive study could be undertaken to follow from the outcomes of the review. There were websites that could not be accessed, and possible resources that may not have been captured by the review. A future study could also undertake interviews and surveys with outdoor workplaces to understand their use of these websites, government and non-government resources and needs with regards to implementing digital interventions to promote sun safety in outdoor workers.

## Conclusion

Digital technology methods are emerging as key tools in communicating skin cancer awareness/prevention messages to outdoor workers/outdoor workplaces. This review provides guidance on further research that is required to understand how these digital interventions can be presented so that they effectively communicate skin cancer awareness and sun protection messages and can be implemented by outdoor workplaces.
